# Transition of Chronic Pediatric Nephrological Patients to Adult Care Excluding Patients on Renal Replacement Therapy with Literature Review

**DOI:** 10.3390/children9070959

**Published:** 2022-06-26

**Authors:** Mirjam Močnik, Sonja Golob Jančič, Nataša Marčun Varda

**Affiliations:** 1 Department of Paediatrics, University Medical Centre Maribor, Ljubljanska 5, 2000 Maribor, Slovenia; sonja.golob.jancic@gmail.com (S.G.J.); natasa.marcunvarda@siol.net (N.M.V.); 2 Medical Faculty, University of Maribor, Taborska 8, 2000 Maribor, Slovenia

**Keywords:** transition, adolescents, nephrology, questionnaire

## Abstract

(1) Background: The transition of children with chronic kidney disease to adult care has become a well-handled issue. However, other patients with normal or mildly decreased renal function also requiring further management and transition are neglected. (2) Methods: A questionnaire was sent to patients with kidney pathology, aged 17 years and older.(3) Results: The patients were mostly high-school (55%) or college students (39%), living with their parents (94%). One third did not know how their disease affected their choice of profession and reproductive health. Furthermore, 46% of the respondents did not know who would continue with their care, and 44% still had a primary pediatrician. (4) Conclusions: A review of the literature on the topic was performed and summarized here. Regular education is the key for successful transfer, not only in chronic kidney and transplant patients, but also in others in whom a decline in renal function can be expected in the future.

## 1. Introduction

Due to the progress in medicine, increasing numbers of children with chronic disease survive into adulthood, which means that there is an increasing need for their appropriate transition from pediatricians to doctors of various specialties as adult patients (hereafter referred to as further treatment or adult caregiver). Pediatric treatment is family-oriented and is based on parental involvement in the decision-making process. By contrast, the treatment of adult patients is patient-centered and requires autonomous and independent decision-making. Transition is important for all adolescents, including those who are not strongly affected by the disease in their daily lives, as it can affect their life expectancy and quality of life. A number of models have been proposed for this transition, most of which suggest starting the transition process between the ages of 18 and 21 [1,2]. In young adults visiting their pediatrician after the age of 21, a delay in transition was noted [3]. In pediatric nephrology, some guidelines propose that the process should start at an even earlier age [4].

Transition involves a comprehensive, developmentally appropriate, and coordinated transition from pediatric treatment to further treatment, which requires a significant amount of patient education. Its goal is to achieve a continuum of care for chronically ill young adults that includes social and emotional development and the acquisition of independent living skills. Transition involves four parties—the patient, his or her family, the pediatrician, and the adult caregiver. At all stages, there are several obstacles that may complicate the process [5,6,7]. A lack of information and fear on the part of patients and their parents have been shown to be the main reasons for late and more difficult transitions [8]. An additional obstacle is the lack of experience with such patients in further treatment. According to research, internists were of the opinion that they need additional education in the field of congenital and childhood diseases, together with the involvement of families [9].

Several models for transition have already been developed, most of which include continuing education, checking the understanding of the disease and its consequences and health effects, self-medication, the understanding of side effects, the impact of drugs on illness and health, the impact of the disease on patients’ ability to work, functionality, reproductive health, and patients’ ability to visit a doctor on their own. Several standardized questionnaires, such as TRAQ, Am I ON TRAC, TR_X_ANSITION Index, are used in practice by pediatricians to assess patients’ readiness for transition [10,11,12,13].

The assessment of readiness for transition in pediatrics is mainly performed on patients with developmental disorders and with chronic pediatric diseases for which further treatment is necessary, such as cystic fibrosis [14] and chronic kidney disease (CKD) of higher grades [4,15,16]. In pediatric nephrology, assessment has also been proposed for patients on dialysis, kidney transplant patients, and patients with rheumatic diseases also affecting the kidneys [15,17].

The goal of our research was to review general readiness for the transition and further treatment of our adolescent patients with various kidney diseases, excluding patients with grade IV to V CKD and renal replacement therapy. We follow a significant number of patients with surgically treated bilateral congenital kidney anomalies with normal renal function and patients with hypertension on non-pharmacological treatment. Insights into how they perceive their readiness offer valuable information for further transition management because these patients also need a follow-up, as they will need medical treatment in the future, as well as transition to adult care. Several studies and recommendations for pediatric patients with CKD and pediatric transplant patients have already been established; however, for most other nephrological patients, such as patients with hypertension, mild CKD, or congenital kidney anomalies, studies and guidelines are scarce [15,18].

## 2. Materials and Methods

A questionnaire about the readiness for the transition was sent to all our patients of appropriate age that were in our care in the year prior to the study. Patients with advanced CKD, transplanted kidney, or on dialysis were not included. The researched group was very diverse and included young adults with chronic kidney disease of stages 1–2 with less severe underlying conditions (congenital anomalies of kidneys and urinary tract, autosomal dominant polycystic kidney disease, glomerulonephritis of different etiology, Alport syndrome, etc.) or with a diagnosis that could lead to kidney damage in adulthood, such as essential hypertension (with some of these patients also needing therapy) or secondary hypertension (for example, due to fibromuscular dysplasia of renal artery). Additionally, we identified young adults with delayed transition and analyzed their answers separately.

The questionnaire included questions drawn from several standardized questionnaires, mentioned above, and included socio-demographic data, questions about the patients’ knowledge about their disease, treatment, its side effects, the effects of drugs and alcohol on the disease, treatment and health, the effect of their disease on reproductive health, profession selection, etc. Next, we asked if they already had a family doctor and if they knew who would be their doctor for further treatment. We asked them about their satisfaction with our facility and their expectations in the future.

The questionnaires were sent by regular mail, and they contained a separate page with the answers that the patients needed to complete. To participate in our study, patients only needed to take a photograph of the last page and send it to an email address created for this purpose.

SPSS Statistics (IBM, version 22) computer statistical software was used to analyze the answers with basic statistical methods. 

Major studies, reports, and reviews on the transition of the chronic pediatric nephrological patients without major decline in renal function were selected. For a comprehensive literature search, multiple databases were included, and an electronic literature search was carried out in MEDLINE via PubMed interface, Google Scholar, and Cochrane Library for all articles published until January 2021. Database-specific search strings were developed and included search terms describing transition in pediatric nephrology, with exception of papers that included chronic kidney disease or transplant patients. A combination of medical subject headings and keywords was used. Titles and abstracts of identified papers were screened by 2 independent reviewers to determine whether they met the eligibility criteria of interest to develop our review. Subsequently, full texts of the remaining articles were independently retrieved by the 2 reviewers for eligibility.

## 3. Results

Of the 220 questionnaires sent, 100 were returned and then included in the study (54 males, 46 females); the participants were aged 19.1 years, on average, with a standard deviation of ±2 years. The youngest participant was 17 and the oldest was 31 years old. On average, they were diagnosed with their illness at 12 years of age (minimum at birth, maximum at 18 years of age), with a standard deviation of 5.9 years, and were followed at our institution for 6.8 years on average (minimum one year, maximum 28 years) with a standard deviation of 5.8 years. In total, 14 patients were aged 21 years or over and, as such, delayed in transition. They consisted of nine students and four employed patients, ten of whom still lived with their parents; three were single and one was in a relationship. The other results are presented in Table 1.

## 4. Discussion

This study evaluates the patients’ self-assessed readiness for transition from a pediatric to an adult health care provider. By understanding the gaps in our education management of transition, we can optimize the transition process. According to research, the majority of patients perceive the ages of 18–19 years and older as the best at which to transfer [8]. In our cohort, the average age was 19.1 years. Mostly, our patients were not delayed in transition, taking the above-mentioned age as an upper limit; however, 14 of the patients were 21 years old or over, with the oldest participant being 31 years of age. As in our study, a considerable number of young adults visiting their pediatrician were noted also elsewhere. Additionally, young adults with chronic disease were more likely to experience delayed care transition [3]. Moreover, most guidelines recommend that patient-adjusted transition can be delayed after the age of 22, especially in children with special needs [18].

The recommendations for the transition of pediatric chronic nephrological patients include individualized approaches upon the completion of physical growth and a joint agreement involving the patient, his or her family, and pediatric and adult nephrology caregivers. Transition should begin in early adolescence (12–14 years) in a gradual manner appropriate to the patient’s developmental stage and intellectual ability. The transfer should occur during a stable phase, after the completion of school education, in coordination with adult caregivers [4].

Growth and development are essential issues for all children and involve a multidisciplinary approach, including support for children and families, which is one of the major differences between pediatric and adult care [4]. The family of the patient is of utmost importance in pediatric care, but quite the opposite in adult care, in which patients are expected to be socially and economically independent. In our study, only six of our subjects were employed and, therefore, economically independent, and only these six were living on their own. In modern society, the age at which people move out of their parents’ home is increasing due to the need for better education and declining living-arrangement prospects [19]. Therefore, although the age of adulthood is 18 in Slovenia, many young adults do not live an independent life and are still largely dependent on their parents. Consequently, in our study, many children do not know what disease they have or the side effects of their medications. In addition, although most of our subjects were already of an adult age, almost half still visited the doctor with their parents. However, most knew which medications they were taking and they took them on their own, showing a certain independence. Importantly, the families of our patients mostly supported their independence, which is not always the case in wider society [20].

We have to stress that half of our patients took no medications, indicating that they might have a milder disease, or a disease for which, at present, no treatment is possible. In fact, we follow a significant number of patients with surgically treated bilateral congenital kidney anomalies with quite normal renal function and patients with hypertension on non-pharmacological treatment, with no medications. However, these patients need follow-up, as they will need medical treatment in the future, as well as transition to adult care. We are of the opinion that plans for transition have to be prepared for all patients with nephrological or urological disease, tailored to the individual, as contended by other authors [4,21].

Much information regarding health and disease status has to be provided when transitioning young patients, and it seems we are the least successful in the field of reproductive health and profession choice. Similarly, Fernandes et al. identified significant gaps in transition education regarding reproductive health and profession choice, but also lacked information about illicit drug use [7]. The knowledge of illicit drug use in our subjects was satisfactory, which may have been the consequence of extensive public health projects in schools and by primary health care providers, providing education on alcohol and drug abuse problems.

Almost half of our subjects did not know who would continue their care in further treatment, which is information we have to provide to guide young adults. In pediatric nephrology, there is a general agreement that patients with CKD stage 3–4 should be transferred to adult nephrologists, whereas those with CKD 1–2 could be followed in primary care and referred to a nephrologist only if there is evidence of progressive kidney damage, hypertension, or proteinuria [4]. Additionally, almost half had still not chosen a family doctor; in Slovenia, this can also be a consequence of a lack of primary care health providers.

Most of our patients were very satisfied with our care and scored us high, which may also be a reason for their delayed transition in some cases. In further care, a similar level of care is expected, which may be a false expectation and may lead to dissatisfaction during further treatment, both for patients and for doctors. Namely, less frequent appointments are to be expected, which is in contrast to the expectations of our subjects. In pediatrics, children are assessed more often to assess their growth, which in adults is already finished. Additionally, in adults, an autonomous approach is expected. Therefore, care for other medical problems should be addressed by the family doctor or other specialist; however, our subjects expect to be counselled by specialists for other problems that are not related to the underlying disease. This is particularly true when further treatment is performed by a family doctor. The fact that more than a third expected a re-interpretation of their illness indicates that the patients’ knowledge about their disease was poor, showing a need for re-education and counselling.

In our cohort, almost a fifth of the subjects will still visit a doctor with their parents in further treatment and a few do not expect to be able to take care of their health on their own. This last fact indicates that many of our subjects are still not prepared for transition and require additional education.

A similar study evaluated patients’ readiness for transition using the TR_X_ANSITION self-assessment tool; the patients were, on average, 21.2 years old, and 75% of them admitted that they were not ready for transfer. The most poorly scored sections in the study were the patients’ self-management skills and knowledge about their disease [22]. A similar study included pediatric patients with inflammatory bowel disease and observed self-management skills in relation to age and the duration of the disease. They concluded that the ability to perform some key transition tasks appears to improve with age, but not with disease duration [23].

In our study, one of the participants additionally wrote that she is developmentally challenged and this is the reason why she feels she cannot take the care on her own. Developmental status is a factor that needs to be considered and taken into account. The transition of patients with neurodevelopmental disability is even more challenging and must be tailored to each individual [24].

Additionally, the subgroup with delayed transition was analyzed. Older patients seem to know more about their disease; they better understand the effects of physical activity, drugs, and alcohol on their health and medications, they go alone to their doctor’s appointments, as they are expected to in further care, and they have more often have a family doctor already. However, also in this group, knowledge about the effects of disease and medications on profession choices and reproductive health is lacking. It is of the upmost importance to clarify these issues, because changes in these areas are expected.

Despite the fact that the problem of transition was recognized at the start of this millennium [5,7], and several recommendations for transition are already in place, a recently published study showed that there is still a lack of awareness and communication among healthcare professionals regarding the transition of young adult patients. According to the study, both pediatricians and providers of further treatment supported a standardized approach; however, the majority of the preparation and support in transitional care was provided by pediatricians, while internists felt of the need to receive formal training related to transition care and were not comfortable caring for young adults with complex medical issues [25].

The optimal transition from pediatric to adult health care also requires knowledge of the psychosocial history of patients with pediatric disease. These patients tend to have a lower quality of life than their healthy peers [26], which is a fact that concerns both pediatricians and adult health care providers. Additionally, for optimal transition, qualified and educated health workers should be provided with knowledge about local culture. Access to care based on patients’ needs should be provided. The timeline of the transition should be individualized based on these needs.

### Literature Review

The need for the continuing care of children with chronic or relapsing kidney diseases was emphasized more than thirty years ago [27]. Although CKD, especially in children undergoing renal replacement therapy, has received much attention with regard to transitioning children to adult services, many other CKD that put patients at risk of rapid kidney-function decline in the future have been neglected to a point. Primary glomerular diseases and congenital anomalies of the kidney and urinary tract are the most common causes of kidney disease in children, with favorable short-term prognosis after clinical remission of the glomerular disease or successful surgical procedure. However, these children demonstrate a significant increase in their risk of future kidney-function decline [28]. Additionally, children with acute renal injury are at risk of kidney damage, which can lead to CKD. The authors support the long-term follow-up of any child who has a severe acute kidney-injury episode [29].

The transition of children with congenital anomalies of the kidney and urinary tract has received some attention from urologists in the last five years, although published studies for other subgroups are mostly lacking. Young adults with chronic urological conditions are often lost to routine medical care in young adulthood and should receive an appropriate transition because of their risk for CKD [21,30,31,32,33,34]. Similarly, other kidney diseases, such as glomerular diseases, tubulopathies, and other rare (hereditary) kidney diseases should be followed and transitioned; however, too often, patients with these conditions are seen by adult caregivers after developing complications [35,36,37,38,39,40,41]. Obesity and obesity-related hypertension are increasing in adolescents, which can lead to cardiovascular events and CKD earlier in adult life. At the point of transition, these at-risk patients should be recognized, followed up, and treated before complications develop [42,43,44]. Patients with urinary incontinence are also a neglected group of adolescents that very often receive suboptimal care in adult life. Their transition and continuity of care should be established [45,46].

We also have to stress that a consensus statement on the transition to adult renal services was published by the International Society of Nephrology and the International Pediatric Nephrology Association, demonstrating the importance of transition for all pediatric renal patients. It should serve as a basic document for transition implementation [4]. A summarized literature review on the topic is shown in Table 2, categorized by subgroups.

## 5. Conclusions

In our study, we assessed the readiness to transition of our chronic nephrological pediatric patients of age, with the exclusion of patients with advanced CKD and patients on renal replacement therapy. We noted a lack of information regarding the knowledge of the diseases the patients had, as well as the impact of their disease on their choice of profession and reproductive health. Further education is needed for our patients, as well as planned, patient-adjusted transitional management, which must be provided to all chronic patients.

This study emphasizes the importance of transitioning pediatric nephrological patients that do not necessarily have regular contact with medical services but could be at risk of further kidney damage necessitating treatment. Therefore, a regular follow-up and transition should be implemented in their care. We recommend the establishment of a transition clinic for all patients of age, with a questionnaire administered prior to their visit, which should involve meeting an adult caregiver. At the same time, the questionnaire should be reviewed by both adult and pediatric parties, with the aim of improving suboptimal areas.

## Figures and Tables

**Table 1:** Results of the questionnaire, presented as N of the checked answers = percent.

Variable	Whole Group (N = 100)	Subgroup with Delayed Transition (N = 14)
Social status	High-school student: 55 College student: 39 Employed: 6	College student: 10 (71.4%) Employed: 4 (28.6%)
Housing status	I live with my parents: 94 I live alone: 5 I am married/living in a relationship: 1	I live with my parents: 10 (71.4%) I live alone: 3 (21.4%) I am married/living in a relationship: 1 (7.1%)
I know what disease I have	Yes: 85 No: 15	Yes: 13 (92.9%) No: 1 (7.1%)
I know what are long-term consequences of my disease	Yes: 86 No: 14	Yes: 12 (85.7%) No: 2 (14.3%)
I know what medications I need to take	Yes: 43 No: 5 I don’t need medications: 52	Yes: 5 (35.7%) No: 2 (14.3%) I don’t need medications: 7 (50%)
I take medications on my own, it is my obligation	Yes: 43 No: 1 I don’t need medications: 56	Yes: 5 (35.7%) No: / I don’t need medications: 9 (64.3%)
I know what are the side effects of the medications I am taking	Yes: 32 No: 15 I don’t need medications: 53	Yes: 5 (35.7%) No: 1 (7.1%) I don’t need medications: 8 (57.1%)
I understand the effect of the drugs and alcohol on my health and medications	Yes: 84 No: 16	Yes: 13 (92.9%) No: 1 (7.1%)
I go alone to the doctor’s appointment	Yes: 58 No: 42	Yes: 13 (92.9%) No: 1 (7.1%)
My family supports my independent care for my health	Yes: 99 No: 1	Yes: 13 (92.9%) No: 1 (7.1%)
I know how my disease affects my profession choice	Yes: 67 No: 33	Yes: 9 (64.3%) No: 5 (35.7%)
I know how my disease affects my physical activity	Yes: 71 No: 17	Yes: 12 (85.7%) No: 2 (14.3%)
I know how my disease and medications affect my reproductive health	Yes: 66 No: 34	Yes: 9 (64.3%) No: 5 (35.7%)
I know who will continue my care in further treatment	Yes, an internist nephrologist: 43 Yes, a family doctor: 8 Yes, other doctor in adult care speciality: 3 No: 46	Yes, an internist nephrologist: 7 (50%) Yes, a family doctor: 1 (7.1%) Yes, other doctor in adult care speciality: / No: 6 (42.9%)
I already have a family doctor	Yes: 55 No: 44	Yes: 12 (85.7%) No: 2 (14.3%)
In your care I was	Very satisfied: 60 Satisfied: 36 Dissatisfied: 4 Very dissatisfied: 0	Very satisfied: 12 (85.7%) Satisfied: 2 (14.3%)
The amount of check-ups and investigations was	Just right: 94 Too much: 6 Not enough: 0	Just right: 14 (100%)
My evaluation of the staff is, from 1 (the least) to 5 (the most)	4,5 in average	4,6 in average
In further treatment I expect	Similar care: 83 Less detailed care: 8 More detailed care: 8	Similar care: 12 (85.7%) Less detailed care: 1 (7.1%) More detailed care: 1 (7.1%)
In further treatment, I will visit the doctor	Alone: 82 With my parents: 18	Yes: 13 (92.9%) No: 1 (7.1%)
In further treatment, I expect (several answers are possible)	Re-interpretation of my illness: 37 Regular and frequent inspections: 61 Counselling for other problems not related to the underlying disease: 54 Frequent blood draws: 22 Frequent ultrasound examinations of the kidneys: 34 Change of therapy: 7	Re-interpretation of my illness: 7 (50%)Regular and frequent inspections: 9 (64.3%) Counselling for other problems not related to the underlying disease: 7 (50%) Frequent blood draws: 3 (21.4%) Frequent ultrasound examinations of the kidneys: 6 (42.9%) Change of therapy: /
In further treatment, I expect to be able to take care of my health on my own	Yes: 98 No: 2	Yes: 14 (100%)

**Table 2:** Presentation of literature review on transition of chronic pediatric nephrological patients from different fields of risk for chronic kidney disease (CKD) development.

Subgroup	Year, First Author, and the Title of the Study	Main Findings
Congenital anomalies of the kidney and urinary tract	2015: Timberlake et al. Identification of adolescent and adult patients receiving pediatric urologic care and establishment of a dedicated transition clinic [30]	Confirms the presence of a sizable population of young adult patients with chronic urologic problems and maturing care needs who continue to receive exclusively pediatric care, and are rarely engaged in preparatory discussions regarding care transition; establishment of a transition clinic to facilitate progression to adult care services at their institution
	2015: Szymanski et al. Current opinions regarding care of the mature pediatric urology patient [31]	Appropriate long-term follow-up of patients with congenital genitourinary conditions is necessary; patients with prior complex surgical reconstruction should be followed by a urologist with specific interest, training, and experience in the area of transitional urology
	2015: Lambert et al. Transitional care in pediatric urology [32]	Children with posterior urethral valves, exstrophy–epispadias complex, cloaca, vesicoureteral reflux, neurogenic bladder, disorders of sex development, cancer, hypospadias, nephrolithiasis, undescended testes, varicoceles, ureteropelvic junction obstruction, solitary kidney, and upper tract anomalies all require long-term evaluation and management
	2016: Bower et al. The transition of young adults with lifelong urological needs from pediatric to adult services: An international children’s continence society position statement [21]	Transitioning and transfer of children with major congenital anomalies requires improved education for doctors and children’s families; early initiation of the transition process should allow the transfer to take place at appropriate times based on the child’s development, as well as environmental and financial factors
	2018: Hettel et al. Lost in transition: patient-identified barriers to adult urological spina bifida care [33]	Patients with congenital diseases are often lost to routine medical care in young adulthood; the decision to pursue adult urologic care is multifactorial; areas for improvement include provider communication at both the pediatric and adult level, as well as education regarding patient preferences and self-readiness
	2020: Yerkes et al. Chronic kidney disease and upper tract concerns after congenital and acquired urinary tract abnormalities: considerations for transition of care in teens and young adults [34]	In individuals with congenital or acquired abnormalities of the urinary tract, there is an inherent risk of CKD, with its associated morbidity and increased mortality risk; the interplay between the upper and lower urinary tract impacts CKD progression; collaborative management between urology and nephrology is highly recommended to address the unique challenges for each individual over their lifetime
Glomerular diseases	2008: Iitaka et al. Transition of children with membranoproliferative glomerulonephritis to adolescence and adulthood [35]	Membranoproliferative glomerulonephritis often continues to adulthood, and patients are usually referred to adult nephrologists; good communication between pediatric and adult nephrologists is important; more in-depth explanation and reeducation about their disease and its management are helpful when these patients reach adolescence to improve their care and help to assure compliance
	2014: Honda et al. The problem of transition from pediatric to adult healthcare in patients with steroid-sensitive nephrotic syndrome (SSNS): a survey of the experts [36]	Importance of transition is highlighted; shifts in steroid dose during transition are a problem in Japan due to the difference in the steroid regimen between pediatric and adult patients with steroid-sensitive nephrotic syndrome
Tubulopathies	2016: Ariceta et al. A coordinated transition model for patients with cystinosis: from pediatrics to adult care [37]	Model of transition as a prototype of children with rare tubulopathies
	2017: Raina et al. Structured transition protocol for children with cystinonsis [38]	Transition has to be structured and depends on four areas of competency: recognition, insight, self-reliance, and the establishment of healthy habits (RISE)
Other rare, hereditary kidney diseases, including neurocutaneous syndromes	2019: Kreuzer M et al. Current management of transition of young people affected by rare renal conditions in the ERKNet [23]	Importance of the transition of these children with transition guidelines; differences in management in different ERKNet centers
	2014: Thiele et al. Transition into adulthood: tuberous sclerosis complex, Sturge-Weber syndrome, and Rasmussen encephalitis [39]	In tuberous sclerosis care, issues tend to evolve from seizure control and development in childhood to renal and psychiatric disease in adulthood; the process of transition/transfer should ensure that these evolving concerns are addressed
	2018: Peron et al. Healthcare transition from childhood to adulthood in tuberous sclerosis complex [40]	Healthcare transition from childhood to adulthood is required to ensure continuity of care; transition for patients with tuberous sclerosis complex is complicated by the multi-systemic nature of this condition, age-dependent manifestations, and high clinical variability, as well as by the presence of intellectual disability in at least half of the individuals; special services and support are required, as is the transition between pediatric and adult teams
	2019: Bar et al. Experience of follow-up, quality of life, and transition from pediatric to adult healthcare of patients with tuberous sclerosis complex [41]	Adult care system needs to develop better multidisciplinary and coordinated care; pediatric-to-adult-health system continuity would decrease the gaps in care, mainly for cognitive and psychiatric impairment
Obesity and hypertension	2006: Doyle et al. Stages of change and transitioning for adolescent patients with obesity and hypertension [42]	Promote self-efficacy and self-care; attention to the promotion lifestyle changes and adherence to treatment regimens as these patients enter adulthood; the use of motivation interviewing and cognitive-behavioral techniques may encourage lifestyle changes and treatment adherence and assist in preparing patients for transition to the adult health care setting
	2013: Shrewsbury et al. Transition to adult care in adolescent obesity: a systematic review and why it is a neglected topic [43]	Internationally, there is an absence of published intervention programs/policies, and limited clinical guidance and expert opinion on the transition of adolescents with obesity
	2019: Zhong et al. Health literacy, nutrition knowledge, and health care transition readiness in youth with chronic kidney disease or hypertension: a cross-sectional study [44]	Health literacy and nutrition knowledge predict self-management and transition readiness and should be evaluated during the transition process
Urinary incontinence	2017: von Gontard et al. Adolescents with nocturnal enuresis and daytime urinary incontinence–How can pediatric and adult care be improved–ICI–RS 2015? [45]	Incontinence in adolescents is a neglected research topic and clinical care is often suboptimal; since patients are seen by both pediatric and adult services, the alignment and harmonization of diagnostic and therapeutic principles is needed; an organized transition process is recommended
	2012: Drake. The adult urology perspective on management of stress urinary incontinence in pediatric urology: ICI–RS 2011 [46]	Regular interaction between the relevant specialties for continuity of care, the best results at transition, the long-term outcomes of pediatric urological procedures, and the development of new surgical techniques

## Data Availability

The full data are available from the corresponding author upon reasonable request.
